# Carotid Sinus Syndrome Secondary to Laryngocele: A Case Report 

**DOI:** 10.22038/ijorl.2020.47421.2586

**Published:** 2021-01

**Authors:** Enzo Chianetta, Barbara Verro, Giuseppe Greco, Rosalia Gargano

**Affiliations:** 1 *Otorhinolaryngology Section, Department of Biomedicine, Neurosciences and Advanced Diagnostic – University of Palermo, Palermo, Italy.*

**Keywords:** Bradycardia, Carotid sinus, Neck mass, Piolaryngocele

## Abstract

**Introduction:**

Carotid sinus syndrome (CSS) is a hypersensitivity of the carotid sinus manifested by atrioventricular sinus bradycardia or decreased arterial pressure of at least 50 mmHg. Triggering factors can be neck movements, shaving of the beard or too-tight collars. CSS can be rarely caused by the presence of malignant or benign masses in the head and neck area.

**Case Report:**

A 49 years-old white woman with a laterocervical mass presented recurrent episodes of sinus bradycardia related to head’s rotation. Neck CT scan revealed a right piolaryngocele and internal left laryngocele. Episodes of bradycardia were disappeared after endolaryngeal carbon dioxide laser assisted marsupialization.

**Conclusion:**

Laryngocele should be sought in the differential diagnosis of patients with bradycardia episodes due to carotid sinus compression. Surgical treatment of laryngoceles can lead to the termination of such episodes.

## Introduction

Carotid sinus syndrome (CSS) is a severe manifestation that can rarely be caused by the presence of malignant or benign masses in the head and neck area (1-4).

The carotid sinus is a dilation of the internal carotid artery in proximity to its origin. The carotid sinus reflex provides to regulate blood arterial pressure. The reflex receptors are located in the adventitia of the carotid sinus and are stimulated by the stretching of the artery wall; its impulses are then transmitted to the medulla by a small branch of the glossopharyngeal nerve called Hering’s nerve. The efferent fibers transmit the impulse through the adrenergic fibers of the sympathetic nervous system and of the vagus nerve towards the AV node, the sinus node and the other blood vessels of the organism (5). CSS is a hypersensitivity of the carotid sinus manifested by atrioventricular sinus bradycardia or decreased arterial pressure of at least 50 mmHg (5); triggering factors can be neck movements, shaving of the beard or too-tight collars (6,7). A laryngocele can be considered an abnormal herniation of the saccule of the larynx. There are three types of laryngocele: internal if this dilation remains within the thyroid cartilage limits, external if it protrudes traversing the thyrohyoid membrane and mixed if laryngocele presents both internal and external components (8). Sometimes, the herniation can be infected and filled of pus: in this case we talk about piolaryngocele. Symptoms include hoarseness, cough, dyspnea, and dysphagia. The treatment of choice is surgical excision (8).

We reported a case of a 49 years old woman who presented internal left laryngocele and mixed right piolaryngocele, causing sinus bradicardia.

## Case Report

**Fig 1:**
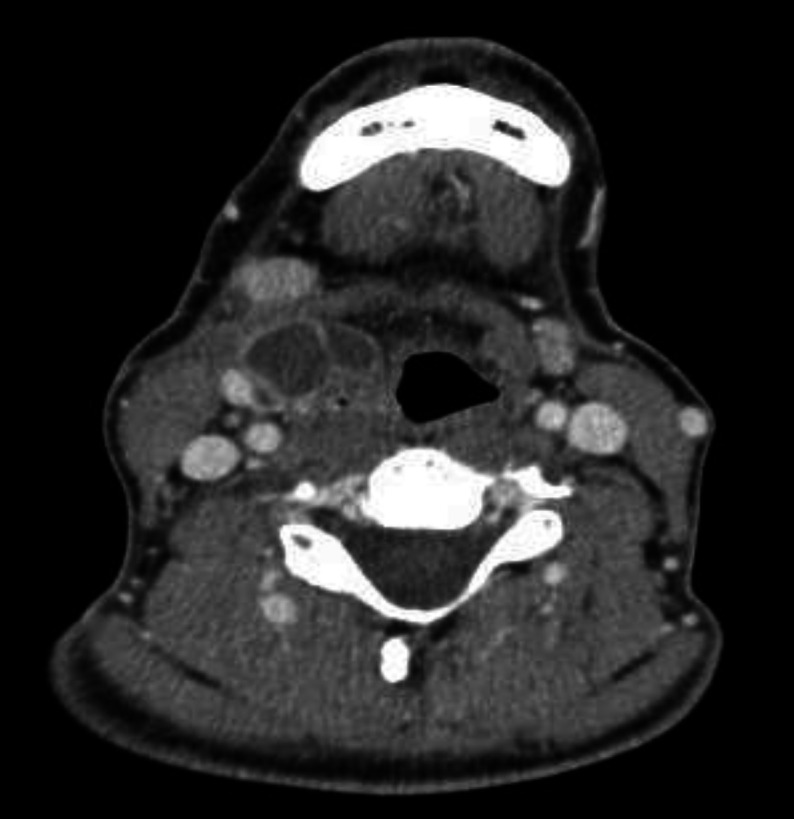
Neck CT scan shows a polylobed collection in right para-laryngeal situs and its anatomical relationships

A 49 years old white woman with a bulky laterocervical right mass, dysphonia and recurrent episodes of dysphagia arrived to our Division for a consult. After medical visit, she was hospitalized for investigation. We performed hematochemical routine tests and neck CT scan before and after contrast medium injection. Hemocromocytometric exam showed an elevated White Blood Cell (WBC) count and slight C-Reactive Proteine (CRP) elevation. The clinical examination showed a 3x2 cm right cervical mass with elastic consistency, hurting at palpation. Optic fibers laryngoscopy reveal oedema of glossoepiglottic fold, arytenoid and false cord on the right side. Neck CT scan showed a fluid polylobed collection with slight parietal enhancement in right para-laryngeal situs (Fig.1). 

This collection extended from laryngeal vestibule to superior margin of thyroid cartilage (3x1.5x4 cm) associated with obliteration of right glossoepiglottic vallecula and omolateral pyriform sinus. Second smaller ovalar mass (1.1 cm of max diameter) revealed in the left anterior para-laryngeal space just under hyoid bone with soft tissue density. There was concomitant right internal jugular lymphadenopathy with max diameter of 11x7 mm.

During hospitalization, we detected a heart rate of 35 bpm in routine monitoring of vital parameters. Serial ECGs were then performed, revealing the presence of sinus bradycardia at 38 bpm on average. Cardiology consultation showed a normal cardiopulmonary examination and hemodynamic stability. Vital parameters and blood tests were normal. The rotation of the head to the left showed a slowdown of cardiac frequency on heart rate monitor without apparent severe bradyarrhythmias. She was subjected to a 24-hours ECG Holter, which showed pure sinus bradycardia. She started the antibiotic therapy with piperacillin/tazobactam and high doses of methylprednisolone with a slight decrement of neck mass. After 10 days of medical therapy without real benefit, the patient underwent to marsupialization and drainage of the purulent collection in endolaryngeal CO2 laser surgery. During the post-operative course, bradycardia disappeared and she repeated 24-hours ECG Holter, that appeared normal.

**Fig 2:**
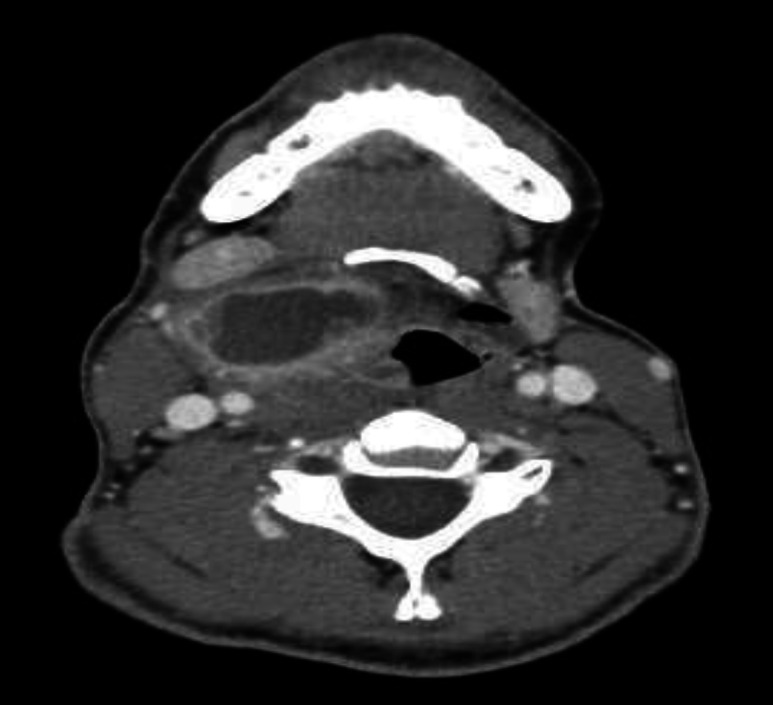
Axial CT scan of the neck during previous hospitalization

Studying the patient's remote pathological history, we found out a previous admission to an ENT Department in 2015 for “suspected right parapharyngeal abscess”. During that hospitalization, the patient presented episodes of sinus bradycardia at 37 bpm on average. Therefore, she was subjected to a cardiological examination that revealed asymptomatic sinus bradycardia. Then, she did a neck CT scan with contrast medium, which showed the presence of a mixed right piolaryngocele (Fig.2). 

She performed therapy with piperacillin/ tazobactam and high-dose corticosteroids and a subsequent surgical drainage of the piolaryngocele, leading to the resolution of bradycardia. Laryngocele marsupialization was not processed, suppurative process, indeed, relapsed.

The cardiologist evaluated past and current medical history and its pre- and post-operative course, confirmed our hypothesis of correlation between the presence of laryngocele and the appearance of CSS with sinus bradycardia due to compression of the carotid sinus.

## Discussion

The carotid sinus is an enlargement located at the origin of the internal carotid artery. The carotid sinus reflex has a role in maintaining an adequate arterial blood pressure. Its receptors are localized in adventitia tunica of carotid sinus and are stimulated by arterial wall stretching (5). Generated impulses are transmitted by a branch of glossopharyngeal nerve called Hering’s nerve to dorsal vagus nucleus of Medulla. Then, the impulse is transmitted to sinus node, AV node and other blood vessel by adrenergic fibers of vagus nerve and sympathetic nervous system (4,9). Carotid sinus syndrome represents a hypersensitivity of carotid sinus manifesting as an AV block, sinus bradycardia or drop in systolic arterial blood pressure greater than 50 mmHg (5). There are 3 types of CSS: the cardioinhibitory response with bradycardia or asystole lasting at least 3 seconds; the vasodepressor response with sudden decrease in systolic arterial blood pressure greater than 50 mmHg; the mixed type (5). Triggers are neck movements, hyperextension of the neck, shaving, tight collars and others similar causes (6,7). In this case, our patient presented bradycardia during left neck rotation. Physiopathology of CSS in patients with neck masses is actually unknown and many hypotheses are elaborated. In particular, compression of carotid sinus by a mass could cause reflex hyper sensitization (10). Muntz et al. postulated that this hyper sensitization could be caused by a mass that compressing the nerve depolarizes its axons (11). Previous studies demonstrated that CSS can be caused by a neck mass that compress carotid sinus. For example, Metha et al. presented a case of CSS caused by metastasis to the left submandibular lymph node manifested by asymptomatic bradycardia (5). Hong et al. reported two cases of CSS caused by the enlarged metastatic lymph node in submandibular regions (4). Other studies reported CSS cases caused by cystoadenolymphoma (12), pleomorphic adenoma and carotid body tumour (13,14). In our case report the patient presented a laryngopiocele that caused sinus bradycardia. Actually, in literature, cases of CSS caused by this lesion are not described. The immediate therapy of CSS includes anticholinergic agents such as atropine, propantheline and others leading to immediate relief of symptoms (15). In our case, anticholinergic drugs were not administered because she presented asymptomatic bradycardia and did not manifest syncopal episodes. Moreover, bradycardia disappeared after surgical excision of laryngocele. Studies, indeed, demonstrated that, in patients with cervical masses, the definitive treatment consists of removing the mass that causes CSS. 

## Conclusion

Our case report demonstrated that whenever patient presents CSS, we should evaluate the eventual presence of laryngocele by laryngoscopy or neck CT scan. This lesion, as other neck masses described in literature, can cause carotid sinus compression. In particular, in our experience, a laryngocele must always be considered because in physical examination it is not always detected as a visible mass due to its small size and can give clinical symptoms only when it undergoes inflammatory processes.
